# YOLOv5 Drone Detection Using Multimodal Data Registered by the Vicon System

**DOI:** 10.3390/s23146396

**Published:** 2023-07-14

**Authors:** Wojciech Lindenheim-Locher, Adam Świtoński, Tomasz Krzeszowski, Grzegorz Paleta, Piotr Hasiec, Henryk Josiński, Marcin Paszkuta, Konrad Wojciechowski, Jakub Rosner

**Affiliations:** 1 Polish-Japanese Academy of Information Technology, ul. Koszykowa 86, 02-008 Warsaw, Poland; wojciechlocher@gmail.com (W.L.-L.); grzetan@gmail.com (G.P.); piotrekhasiec@gmail.com (P.H.); mpaszkuta@pja.edu.pl (M.P.); konrad.wojciechowski@pja.edu.pl (K.W.); 2 Department of Computer Graphics, Vision and Digital Systems, Silesian University of Technology, ul. Akademicka 16, 44-100 Gliwice, Poland; adam.switonski@polsl.pl (A.Ś.); henryk.josinski@polsl.pl (H.J.); 3 Faculty of Electrical and Computer Engineering, Rzeszow University of Technology, al. Powstancow Warszawy 12, 35-959 Rzeszow, Poland; tkrzeszo@prz.edu.pl

**Keywords:** drone detection, unmanned aerial vehicle, deep learning, motion capture, Vicon, YOLO, drone localization

## Abstract

This work is focused on the preliminary stage of the 3D drone tracking challenge, namely the precise detection of drones on images obtained from a synchronized multi-camera system. The YOLOv5 deep network with different input resolutions is trained and tested on the basis of real, multimodal data containing synchronized video sequences and precise motion capture data as a ground truth reference. The bounding boxes are determined based on the 3D position and orientation of an asymmetric cross attached to the top of the tracked object with known translation to the object’s center. The arms of the cross are identified by the markers registered by motion capture acquisition. Besides the classical mean average precision (mAP), a measure more adequate in the evaluation of detection performance in 3D tracking is proposed, namely the average distance between the centroids of matched references and detected drones, including false positive and false negative ratios. Moreover, the videos generated in the AirSim simulation platform were taken into account in both the training and testing stages.

## 1. Introduction

The popularity of drones has increased significantly in recent years. One of the main reasons for this is the advancement of technology, which has led to smaller, more affordable, and user-friendly devices. This allows them to be used in both professional and amateur applications [1]. The miniaturization of sensors and cameras has made it possible to equip drones with high-resolution cameras, GPS, and other sensors that enable them to collect data and perform tasks that were previously impossible or very difficult to achieve. Thanks to that, they can be used to survey large land areas, inspect buildings and structures, and even deliver packages [2].

The increasing popularity of drones also has its downsides. There is a potential risk to privacy, as they can be used to gather sensitive information or to spy on people without their knowledge or consent [3]. Additionally, they can also pose a risk to air traffic, as they can collide with other flying objects or distract pilots. Furthermore, drones can be weaponized and used for terrorist attacks, smuggling of illegal goods, and espionage [4]. Therefore, it is essential to develop drone detection mechanisms to minimize these risks.

Actually, detecting drones is quite a challenging task because they are small, lightweight, and can fly at high speeds, which makes them difficult to spot against complex backgrounds such as trees or buildings [5]. Additionally, they can fly at high and very low altitudes, making them hard to detect using radar and other sensors. Currently, there are many methods to detect drones, but they are often not accurate enough and expensive.

One group of drone detection methods is the use of radar systems and sensors [6,7]. These methods have some benefits but some limitations as well. For example, radar can be jammed [8] or blocked by buildings, or sensors can have difficulty distinguishing drones from other small flying objects such as birds, making it difficult to detect drones accurately [9]. Moreover, these solutions require additional detectors and sensors, which makes them more expensive.

**Figure 1:**
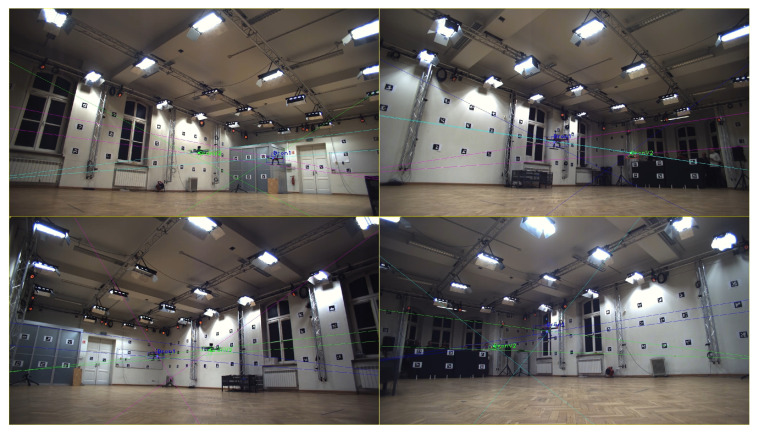
Multi-camera drone registration. Determined bounding boxes and epipolar lines are visualized.

Primarily, 3D drone tracking is of high practical relevance, for instance, for the game industry or for security systems [10]. This means that the 3D positions of the flying drones in successive time instances are determined. Their 3D tracking can be implemented on the basis of multi-camera video registration, as visualized in Figure 1. If the drones’ positions are known, along with internal and external camera parameters, it is possible to reconstruct the 3D coordinates. In the case of multiple drone tracking, object matching on different images has to be performed using, for instance, epipolar geometry or a selected optimization technique. The first and crucial stage of such a system is drone detection on 2D images, in which bounding boxes are determined. If properly found, their centers correspond to the mean 2D position and are taken for further 3D reconstruction.

Recently, detection methods on images using neural networks have gained significant popularity [5,11]. One of the main reasons for that is their high accuracy and speed of detection. Their efficiency is caused by the fact that they can be trained to identify drones in a wide range of conditions and environments. This is because training datasets can be populated with data containing instances that make detection difficult [12]. These include changes in lighting conditions, the drone disappearing from the camera’s field of view, changes in the angle at which the drone is seen by the camera, and many more. Additionally, neural networks can be updated and retrained as new models are released, which allows them to adapt to new objects.

Currently, there are many solutions using neural networks for object detection. Each of these approaches has distinctive features that make it possible to select the right solution for the requirements of a given task. For example, YOLO [13] is an object detection algorithm that predicts bounding boxes and class probabilities in a single pass, making it ideal for real-time detection. On the other hand, Densenet201 [14] excels in image recognition tasks, focusing on high accuracy rather than real-time performance. It is characterized by its dense connectivity, where feature maps from preceding layers are concatenated to the current layer. This design promotes feature reuse and enables efficient gradient flow during training. Another technique could be background subtraction with MobileNetV2 [15]. It focuses on detecting objects in dynamic scenes by first extracting foreground objects using background subtraction and then applying MobileNetV2 for object detection. This approach can be useful in scenarios where object motion plays a significant role, but it is slower than the YOLO algorithm. There are advantages and disadvantages to each of these methods, so a series of tests should be conducted to identify the right one for the given task.

The far-reaching goal of the presented research is related to the construction of a 3D drone tracking system operating on multi-camera video registration. The main application of the mentioned system is its use in augmented reality systems. These systems will operate in closed spaces like sports halls, hence the need to also ensure the safety of the drones so that they do not collide during flights with multiple drones in a limited confined space. In order to perform tracking in three-dimensional space, drones must first be detected; hence, this work is focused on precise drone detection in 2D images. The proper technique is selected, trained, and evaluated based on both simulated video sequences generated in the AirSim environment and real multi-camera video data with synchronized, multimodal reference data acquired by the Vicon system [16]. New algorithms needed to be developed to determine the accurate position of the drone in a 3D space. The contribution of this paper focuses on the following elements:

- The use of markers to create a different asymmetrical cross for each drone for 3D position reconstruction to enable the accurate localization of drones in a 3D space.
- The development of a projection algorithm from 3D to 2D for multiple cameras, which enables the precise and automatic labeling of a real dataset.
- The proposition of custom metrics (Mean Centers’ Distance, MCD) for assessing the quality of drone detection based on a comparison of bounding box centers.
- Preparation and sharing of databases containing simulation and real data for deep neural network training and testing.
- Analysis of speed and precision for multiple YOLOv5 settings.
- Comparison of the performance of YOLOv5 and YOLOv8 on our datasets.

The structure of this article is as follows: Section 2 contains a review of the literature in terms of drone detection using deep neural networks. The next section (Section 3) presents information about the prepared dataset. Section 4 contains the description of the utilized methods. The experimental results are presented in Section 5. Finally, the summary and conclusions are given in Section 6.

## 2. Related Work

The most widely discussed drone detection methods in recent research related to deep neural networks are those using the YOLO (You Only Look Once) algorithm. YOLO is a popular method for detecting objects due to its speed and efficiency. Its real-time performance, high accuracy, and simplicity make it suitable for a wide range of applications. The authors of [17] applied YOLOv4 to detect drones and distinguish them from birds. The task was challenging due to the high similarity of the detected objects. The authors used about 10,000 images to train the neural network. Analyzing the public portions of the dataset, it can be noticed that most of the images contain drones in the foreground of the scenery. This, combined with the detection threshold of about 0.35, allowed the authors to obtain high-accuracy results of 83% and a mAP of 84%. An analogous application of YOLOv4 is described in [18], where training using a neural network was performed on similar images in terms of complexity, but the dataset itself contained more than half as many images as in the previous case. This contributed to about a 10–20% worse accuracy than in the previously discussed work. A distinguishing feature of this work is that the model was also tested on their proprietary dataset that consisted of footage taken during a drone flight. With the footage, the authors were able to determine the model’s processing efficiency, which was around 20 fps. However, the comparison to other solutions is difficult, as it depends on both the GPU used for the computations and the model itself. The described papers show the YOLOv4 algorithm to be promising for drone detection; however, in our case, we need more accurate detection results carried out under heavier scenery conditions.

Another approach was presented by the authors of [14]. They used the Densenet201 neural network to detect three types of drones. The neural network was trained using a synthetic dataset created by the authors. For this purpose, they developed a data generation method that made it possible to produce synthetic data with labels in a short time. To test the model, they used footage of the drone’s flight, which often showed scenery where the drone was depicted in the central part of the image. The presented method achieved an accuracy of 92.4% and a mAP of 88.8%. Although the tests were performed using recordings, the results of the detection speed were not shown. Despite fairly high detection accuracy scores, the algorithm is too slow for real-time drone detection using several Full-HD cameras simultaneously.

This paper [19] demonstrates that the MobileNetV2 convolutional neural network can provide comparable results in drone and bird detection to those previously discussed. What distinguishes their approach is the division of the detection process into two separate stages. First, the detection of moving objects based on background subtraction [20] is performed, and then the classification of drones and birds is carried out using the convolutional neural network. This method has one major limitation related to the fact that the moving background strongly affects the detector’s performance. The aforementioned method allowed the authors to obtain a mAP score of 70% using a threshold of 0.5. In contrast, the detection speed was only 9 fps, which may be highly dependent on the GPU. The complexity of the operations needed to perform detection is too high to allow for the real-time processing of video from several Full-HD cameras.

In [21], the performance evaluation of the 2D object tracking registered by the drone’s camera is based on Vicon measurements. This is a different problem than that of 3D drone tracking faced in this paper. Moreover, the results are presented in a graphical form, without providing any quality measures.

Although there are many publications discussing drone detection, there is still a lack of research presenting well-tested solutions in challenging conditions with reliable performance evaluations for the purpose of 3D tracking. In this paper, a novel YOLOv5 deep convolutional neural network, hybrid data simulated in the AirSim Platform, and multimodal data registered by the Vicon system are used. This made it possible to automatically create a large number of labeled data, which are applied to train the neural network and perform detailed tests of the model’s performance and precision.

It is worth paying attention to the need to verify machine learning systems, in particular neural networks, using formal methods based on mathematical foundations. The authors of [22] reviewed the categories of formal methods (abstract interpretation, semantic static analysis, model checking, proof assistants, deductive verification, model-based testing, and design by refinement) and briefly described the methods used for neural networks, decision tree ensembles, and support vector machines. It should be emphasized that this review was not limited to already-trained models, but also included the phases of data preparation and model training, often omitted in these type of studies. Particular attention was paid to the problem of local robustness against adversarial perturbations. For convolutional networks, the CNN-Cert framework was referred to, the mathematical foundations of which are given by [23]. The next consistent step in the application of formal methods is the verification of an ML-based complex system-of-systems, which is considered by [24], illustrating their approach with an example based on the flight of autonomous UAVs in formation.

## 3. Dataset Preparation

In the training and testing of the YOLOv5 network, primarily real and multimodal data registered in the Human Motion Lab (HML) of the Polish-Japanese Academy of Information Technology are used. Moreover, they are extended by simulated video sequences generated in the AirSim Platform. All the data are freely available at http://bytom.pja.edu.pl/drones/ (accessed on 10 July 2023).

**Figure 2:**
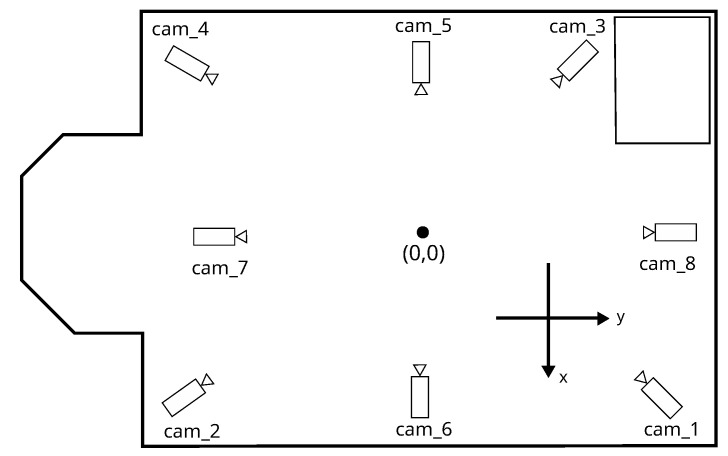
Top view of the laboratory scene with the position of cameras.

The summary of captured sequences containing numbers of cameras and tracked drones as well as video resolution, duration, and frames per second are presented in Table 1. The spatial arrangement of cameras is depicted in Figure 2. The synthetic datasets contain videos from eight cameras, while the real ones contain data from only four corner cameras.

### 3.1. Real Multimodal Data

In the measurements, the Vicon system was used as the gold standard. It provides a motion capture acquisition based on a set of infrared cameras that reconstruct the 3D positions of moving markers. Moreover, the system is equipped with four calibrated RGB cameras synchronized with the motion capture registration. It is capable of capturing high-precision multimodal data at high frame rates and can track multiple objects or people simultaneously. The Vicon includes a software suite that allows users to process and analyze the motion capture data and export them to well-known formats. These properties made Vicon an ideal solution to train and validate detections performed by neural networks.

**Figure 3:**
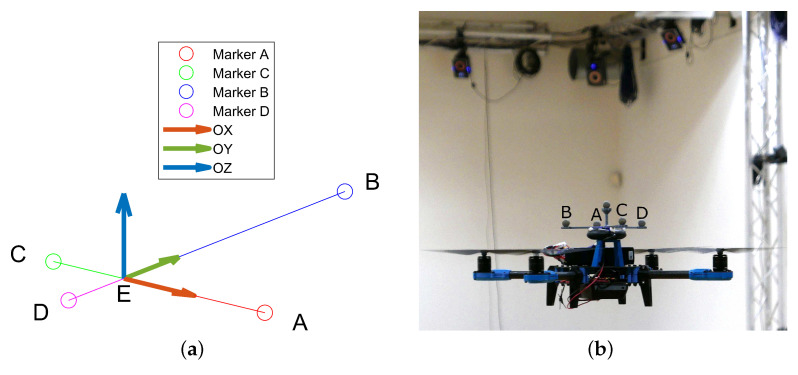
Markers forming asymmetric cross attached to a drone. To distinguish the drones from each other, each cross is different. (**a**) 3D model with local coordinate system XYZ. (**b**) Example of a drone.

To achieve this, markers are attached at the top of tracked drones. They form an asymmetric cross structure with known proportions, as visualized in Figure 3.

A motion capture system computes their 3D coordinates in subsequent time instants, which allows for the determination of the 3D drone position and orientation in the following way: In the first stage, the center of the cross is established, and markers are identified. This is realized by comparing distances between the markers. Due to the limited precision of the measurements, we cannot assume that the arms of the cross are coplanar. Thus, we compute the position of the center marker as the projection of point *B* from Figure 3a on the AC line segment. Then, versors of the local drone coordinate system XYZ are determined using the following formulas:

(1) $$\hat{x}=\frac{\vec{EA}}{\left|EA\right|}$$

(2) $$\hat{y}=\frac{\vec{ED}}{\left|ED\right|}$$

(3) $$\hat{z}=\hat{x}\times \hat{y}$$

Having information concerning the *i*-th drone size; the 3D coordinates of bounding box vertices $V_{l}^{i}=\left(V_{l,x}^{i},V_{l,y}^{i},V_{l,z}^{i}\right)$ in the local system XYZ; its reference point—the origin of the local system, which is the center *E* of the cross; as well as the plane orientation pointed by versors $\hat{x}$, $\hat{y}$ and $\hat{z}$, the 3D coordinates of the vertices $V_{g}^{i}$ in the global system can be calculated.

(4) $$V_{g}^{i}=V_{l,x}^{i}\cdot \hat{x}+V_{l,y}^{i}\cdot \hat{y}+V_{l,z}^{i}\cdot \hat{z}+E$$

**Figure 4:**
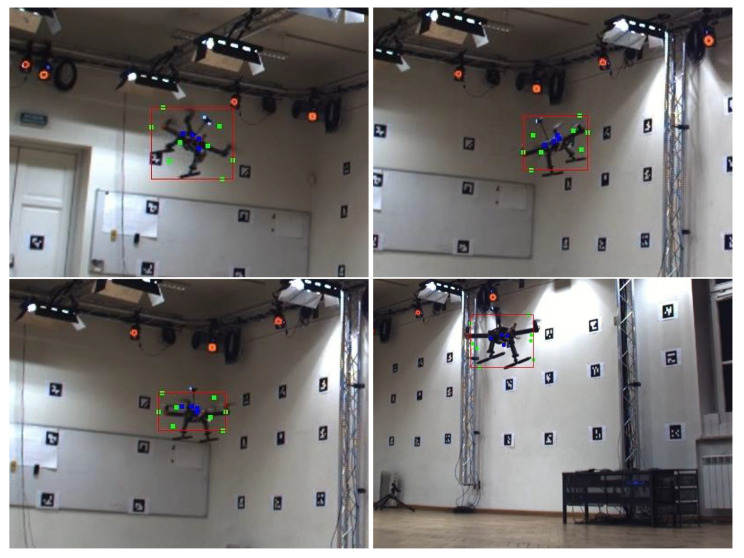
Bounding box extraction based on the motion capture measurements. Markers and 2D and 3D bounding boxes are labeled with blue, green, and red colors, respectively.

Finally, the vertices of the 3D bounding box are projected on the 2D image based on internal and external camera parameters. Thus, we obtain a 2D bounding box, being the ground truth data for the neural network. The process is visualized in Figure 4, in which markers of the motion capture system are labeled in blue, vertices of the 3D bounding box in green, and extracted 2D bounding box in red.

**Figure 5:**
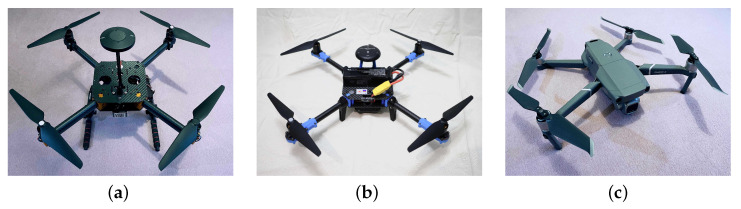
Physical drones used in the dataset. (**a**) Custom made. (**b**) X500 V2. (**c**) DJI Mavic 2.

Three separate recordings, each with a different flying quadrotor drone, were used for training and testing. The selected models are a custom-made one with the CUBE Orange flight controller (Figure 5a), an “X500 V2”-based one with HolyBro 6C (Figure 5b), and a DJI Mavic 2 (Figure 5c). In total, around 18 000 frames were obtained using four calibrated and synchronized RGB cameras with Full HD, 25 fps.

### 3.2. Simulated Data

To generate simulated data, the AirSim [25]—an open-source, high-fidelity platform for preparing animation with autonomous systems, including drones, cars, and other vehicles—was chosen. It uses the Unreal Engine and provides realistic physics and sensor models for accurate simulation with reference ground truth data.

**Figure 6:**
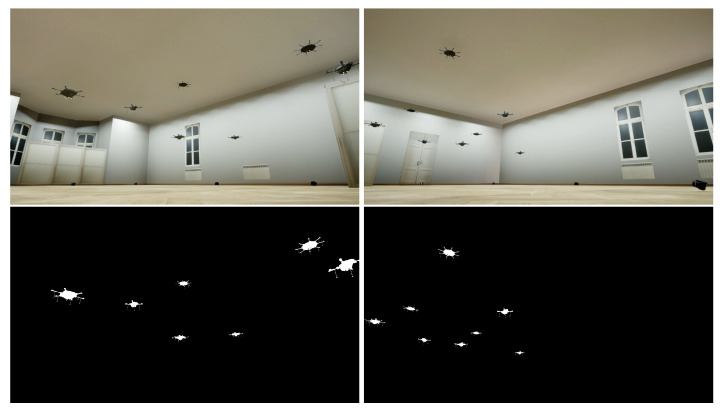
AirSim simulation with eight flying drones. Two selected video frames (**at the top**) with corresponding drone masks (**at the bottom**), were obtained at the same time from different cameras.

We replicated the coarse appearance of the HML laboratory in the virtual reality in which our simulations were located. The setting with eight cameras performing 2D projections was applied. This means that for every animation, eight video sequences were obtained. Due to the animation created, the 3D positions of the drones were known. This makes it possible to extract 2D masks and bounding boxes processed by the neural network. An sample simulation frame with the corresponding drone masks, viewed from the perspective of two selected cameras, is depicted in Figure 6.

**Figure 7:**
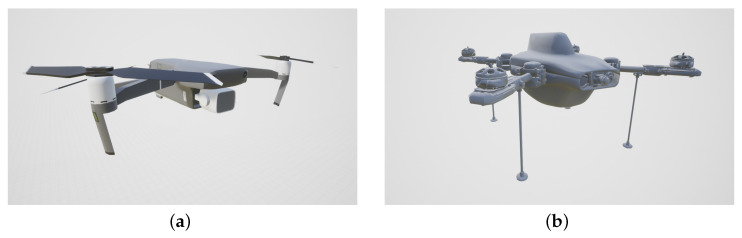
Virtual drones used in the dataset. (**a**) DJI Mavic 2. (**b**) Custom designed.

Ten simulations with a different number of flying drones (2–8) and 20 s of duration were prepared. A DJI Mavic model 2 (Figure 7a) and a custom-designed one (Figure 7b) were used in the animations. Eight virtual camera streams were created with 25 fps each, providing a total of about 40,000 synthetic images for training and testing.

## 4. Methods

For the purpose of drone detection, the YOLOv5 architecture was chosen. To enlarge the training dataset and make it more representative, data augmentation was carried out. In the performance evaluation, the classical mAP measure as well as the one designed to be more adequate for assessing the usability of the detection in 3D object tracking were applied. Some details are described in the following subsections.

### 4.1. YOLOv5

The high performance of deep neural networks in image recognition is caused by the availability of huge datasets and extensive computational resources. The generalization of their work is achieved by the multilayer architectures containing convolutional and fully connected layers. The neuron weights are updated by the gradient descent procedure and the backpropagation algorithm.

One of the most popular algorithms using convolutional neural networks for object detection in images and videos is YOLO (You Only Look Once). Among other applications, it has been used in face mask recognition [26], object detection on drone-captured scenarios [27], and heavy goods vehicle detection [28]. What sets it apart from most other solutions is its performance. It is a single-shot algorithm, meaning that it makes predictions for all objects in an image or video frame in a single pass. This makes it well-suited for real-time object detection on video, where speed is critical.

**Figure 8:**
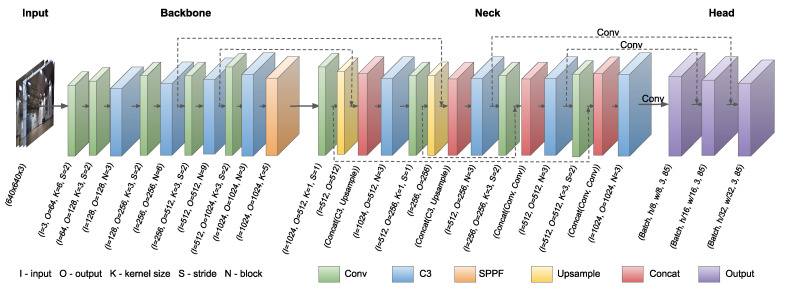
Simplified architecture of the YOLOv5.

The YOLOv5 version that uses PyTorch instead of the Darknet framework was selected. The network structure contains three main components: a backbone, a neck, and a head, as shown in Figure 8. In the backbone, the new CSP-Darknet53 architecture was applied. It uses the C3 layer, which is a simplified version of the used CSP Bottleneck layer, by removing one of the four main convolutions from inside of CSP Bottleneck layer. To reduce the number of parameters, truncation of the gradient flow is performed. The CSP networks preserve DenseNet’s feature reuse qualities and reduce the redundant gradient information that normally occurs, which helps to increase the inference speed [29]. In the neck block, a modified version of the PANet (Path Aggregation Network) with C3 layers and the SPPF (Spatial Pyramid Pooling Fast) have been used [30]. The SPPF is an improved, faster version of the popular SPP with an increased flow of information, making it easier to locate pixels correctly. The head block is the same as the one used in YOLOv3 and YOLOv4, which contains three convolution layers that are used to predict the location of bounding boxes and calculate the scores. In our case, the head block was modified by performing the transfer learning, which starts the training with pre-trained weights achieved for the COCO dataset. The transfer learning may result in a less precise network, which is originally adapted to a different detection problem. However, in most cases, it allows us to obtain satisfactory results with fewer training samples and with a lower computational cost and minimize the probability of network overfitting.

### 4.2. Data Augmentation

**Figure 9:**
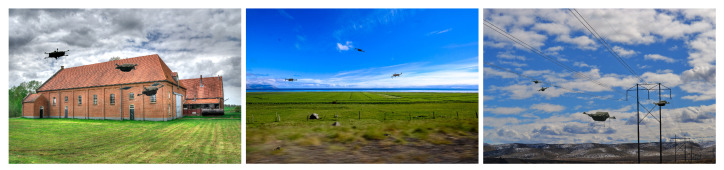
Examples of synthetic images.

In order to avoid overfitting, the training process has been enhanced by synthetic images. Primarily, two base datasets were prepared. The first one contained about 300 images with drones cropped from the randomly taken video frames. In the case of the HML recordings, this was performed manually using graphics utility, while for AirSim simulations, it was performed automatically on the basis of the generated masks (see Figure 6). The second dataset contains background images. We combined both of them by randomly placing drones in background images. Moreover, some extra affine transformations were applied, and some examples of such synthetic images are shown in Figure 9.

Furthermore, data augmentation was employed on-the-fly, together with the loading of the dataset for neural network training. As in the previous case, a set of transformations provided by the Albumentations library [31] were implemented. Techniques such as random cropping (30%), resizing (20%), flipping (50%), noise (20%), and brightness (60%) were applied randomly to each image with indicated probabilities.

### 4.3. Evaluation Metrics

The classical measure for the assessment of the performance of the detection represented by bounding boxes is mean average precision (mAP). It is based on the intersection over union (IoU) ratio calculated for the matched predicted and reference regions. For the given confidence value of the recognized object by the YOLO network, the IoU is thresholded to determine the true and false positives as well as false negatives. Thus, precision (positive predictive value) and recall (true positive rate) measures can be computed. Analyzing different confidences, the area under the precision/recall curve, called average precision, is calculated. To accomplish this task, eleven-point interpolation method is selected, where eleven points are evenly placed on the recall axis, and the average of corresponding precision values is computed. Moreover, to obtain a monotonic curve, smoothing is carried out in such a way that every precision value is substituted by the maximum calculated for recalls equal to or greater than the current one. Finally, mean average precision is determined as the mean obtained by different thresholds applied for the IoU ratio. In a default variant, nine uniformly distributed values in the range (0.1,0.9) are taken into consideration. In the more restrictive one, only the range (0.5,0.9) is analyzed, which means that detections covering less than 50% of the reference objects are rejected and treated as false.

However, from the point of view of 3D object tracking in which 3D coordinates in the global system of the central point are reconstructed, the measure analyzing the IoU ratios of the detected and reference bounding boxes is far from perfect. This is mostly due to the fact that drones usually have some small vertical elements like antennas, landing legs, etc. (see Figure 5a), which, when included in the bounding box, shift its center from the center of the base. On the other hand, when they are not included in the bounding box, they make the bounding box smaller than that of the reference, and the IoU is low even though the drone’s position may be perfectly matched. Therefore an alternative approach in the form of Mean Centers’ Distance (MCD) has been proposed in order to achieve a better quality of drone tracking, which will be the main goal of the future study. It calculates the mean distance between centers of bounding boxes, processed in the successive stages of the 3D reconstruction. Similarly to the mAP measure, it requires matching detection with the closest ground truth object; however, instead of IoU values, now it is based on centers’ distances. Considering the situation in which there are more references or detected objects, false negatives and positives may occur. To determine them, a distance threshold is applied in order to reject too-distant matches. Unmatched detected and reference objects are considered false positives and negatives, respectively. The threshold value influences the measure calculated, and it should be chosen depending on the expectations. If a shorter mean distance is demanded, the lower threshold must be selected, but the side effect is a greater number of false positives and negatives. To visualize these relationships, graphs, determined by applying different distance thresholds, are presented.

It is more convenient to interpret false positives and negatives if they are expressed on a relative scale rather than an absolute one. In the classical variant, this is achieved by normalizing them by the total number of negatives and positives, respectively. However, in the faced problem, the true negatives are unknown. Thus, we divide both measures by the number of all positives. The resultant values are called false positive (FPR) and negative (FNR) rates.

## 5. Results

To assess the detection performance, disjoint training and testing sets were prepared. In the preprocessing stage, images are scaled, preserving the aspect ratio to match the input resolution of the neural network. In the postprocessing phase, they are upscaled back to their original Full-HD resolution. Moreover, pixel values are standardized. Overlapping and resultant bounding boxes are eliminated on the basis of a non-maximum suppression procedure. Quality measures are calculated, and their analysis is conducted separately for the AirSim and HML data.

In Table 2, the mAP values for the default (0.1,0.9) and more restrictive (0.5,0.95) IoU ranges, obtained by the neural network operating on images with default resolution 640×640, are depicted. The detections are pretty accurate only for the AirSim sequences with mAP values greater than 0.8. They are much less precise for the HML videos. In particular, the mAP@0.5:0.95 is very low, which means that most detections have an IoU ratio below 0.5.

Because of much worse performance in the case of the HML data, additionally, higher resolutions (736×736 and 1024×1024) of the images processed by the YOLO network were investigated. Despite much greater computational expansiveness, the obtained recognition improvement is insignificant.

**Figure 10:**
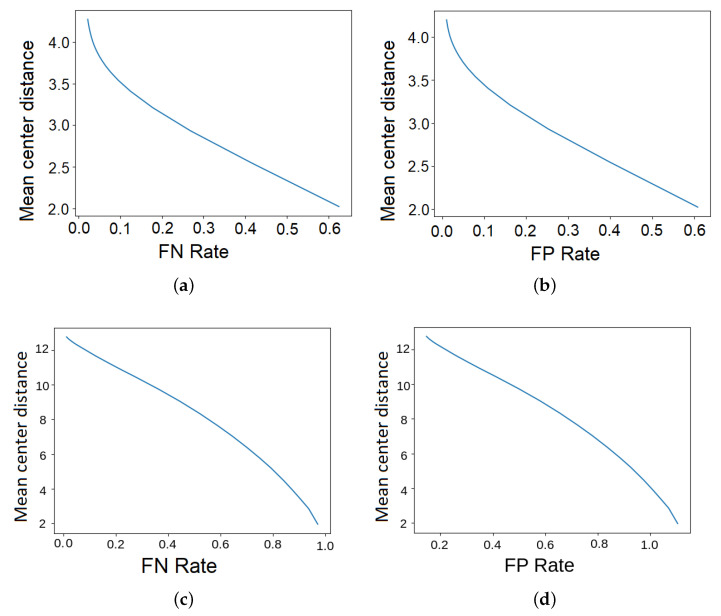
Mean Centers’ Distance in relation to false positive and negative rates for the AirSim and HML data. The resolution of the processed images by the YOLO network is 640×640. (**a**) AirSim, MCD/FNR. (**b**) AirSim, MCD/FPR. (**c**) HML, MCD/FNR. (**d**) HML, MCD/FPR.

The results of the achieved Mean Centers’ Distance and false positive and negative rates for matching distance thresholds 10, 20, and 30 are presented in Table 3, and complete dependencies between MCD, FNR, and FPR are visualized in Figure 10. The distances are expressed in image pixels in Full-HD resolution. Once again, much better results are obtained for AirSim data. The drones are detected with only 3–4 pixels of mislocation. The false negative and positive rates are insignificant—a tiny percentage of detected and actual objects remains unmatched. It is more troublesome for the real videos recorded in the HML lab. MCD, FNR, and FPR measures are higher, which means mislocations are greater, and the number of undetected and incorrectly recognized drones is larger. In spite of that, the results are quite satisfactory for HML data as well. The average and median lengths of the reference bounding boxes’ diagonals are 270 and 210, respectively. This means that the highest mean relative mislocation for matching threshold 30, which corresponds to acceptable false rates lower than 13%, is only about 5.5% or 7% percent.

The results obtained for the sizes of the processed images 640×640 and 736×736 are pretty similar. Surprisingly, for the highest resolution 1024×1024, the least-accurate drone detection is achieved, even though it gives the best coverage of the matched bounding boxes, as described by the mAP values from Table 2.

The balance between the expected Mean Centers’ Distance and one of two remaining metrics (false positives or false negatives) can be influenced, as presented in Figure 10, which is particularly useful for the HML data. For instance, the reduction of the mean drone mislocation below ten pixels causes approximately 35% of drones to be undetected and 45% of detections to be rejected.

The large differences in detection results between the AirSim and HML recordings led us to test a different neural network architecture. For this purpose, YOLOv8 models were trained using the datasets described earlier. The detection results of the new models compared to those previously obtained using YOLOv5 are shown in Table 4. Similar to YOLOv5, the results for YOLOv8 were significantly better when tested on AirSim recordings than when testing on HML. The mAP results were found to be slightly better for YOLOv8. However, the MCD results for HML, which are more relevant in this case, proved to be worse than for YOLOv5. The bounding box centers were fitted about 1px worse than was the case with YOLOv5; in addition, the FNR and FPR metrics using a threshold of 10 and 20 showed worse results, which could significantly hamper further work.

## 6. Summary and Conclusions

In this paper, the problem of drone detection on 2D images as the first stage of a 3D object tracking system is presented, where YOLOv5 network and transfer learning are applied to accomplish the task. In the training and validation, simulated data prepared in the AirSim Platform, as well as real multimodal data registered in the HML lab, are used. To automatically label real video sequences, the method of establishing the 3D drone orientation based on the positions of the markers of the asymmetric cross is proposed. In the evaluation, the classical mean average precision as well as the measure that determines the mean distance between matched bounding box centers are used.

The obtained results are impressive for the AirSim video sequences and are still acceptable for the HML ones. Primarily, the difference may be explained by a much larger simulated dataset in comparison to the real one. Moreover, as seen in Figure 1, Figure 4 and Figure 6, a simpler background was prepared in the AirSim, and simulated data are not influenced by any acquisition noise, which makes the detection easier.

It was expected that we would achieve greater improvement in the detection when the images processed by the YOLO network were of higher resolution; however, there was only a slight improvement reflected by mAP values. Still, as explained in this paper, the mAP does not fully correspond to the measure that compares drone centers, which is of greater importance in the case of drone tracking. The insignificance of improvement may have been caused by a greater number of parameters of the network when processing images with higher resolutions, which suggests that the learning is more demanding and may require a larger training set.

Similar tests were performed on the newest YOLOv8, where significantly better results were expected than when using the YOLOv5 model. In the example described in this paper, the tests showed YOLOv8 was advantageous for the MCD metric only for the AirSim dataset. Based on the results of the HML recordings included in Table 4, which are more important in our case due to the target use of the algorithm in real-world conditions and because the detection times of the individual models were very similar, YOLOv5 was selected for further work.

However as mentioned before, even the results obtained from the HML data are still promising from the point of view of 3D object tracking, which is our next challenge, as the distances are relatively small. Moreover, we can control the matching between detections on different cameras in a quite analogous way as in the proposed evaluation procedure. By applying the threshold for distances to epipolar lines, we can expect to achieve similar dependencies as the ones presented in Figure 10.

## Figures and Tables

**Table 1:** Summary of collected datasets.

Dataset	#Drones	#Drones Models	Duration [s]	Flight Pattern
Synthetic data, 1920 × 1080@25fps, 8 cameras				
AirSim_1	4	1	10	horizontal
AirSim_2	10	1	10	synchronized
AirSim_3	10	1	10	synchronized
AirSim_4	8	1	20	synchronized
AirSim_5	6	1	20	synchronized
AirSim_6	8	1	20	asynchronous
AirSim_7	8	3	20	synchronized
AirSim_8	8	3	20	sinusoidal asynchronous
AirSim_9	8	3	20	sinusoidal asynchronous
AirSim_10	2	1	20	free
Real data, 1924 × 1082@25fps, 4 cameras				
HML_1	1	1	78	free
HML_2	1	1	60	free
HML_3	2	1	90	free

**Table 2:** Mean average precision for IoU ranges (0.1,0.9) (mAP@0.1:0.9) and (0.5,0.95) (mAP@0.5:0.95) obtained in the AirSim and HML validation with a different resolution for the processed input image.

	Resolution	mAP@0.1:0.9	mAP@0.5:0.95
AirSim	640×640	0.89	0.83
HML	640×640	0.41	0.09
	736×736	0.43	0.11
	1024×1024	0.44	0.12

**Table 3:** MCD, FNR, and FPR for AirSim and HML validation with a different resolution of the processed input image, obtained for various threshold values applied in the matching procedure.

Threshold	Metrics	AirSim	HML		
		640×640	640×640	736×736	1024×1024
10	MCD	3.71 px	6.78 px	6.74 px	6.86 px
	FNR	0.07	0.78	0.77	0.79
	FPR	0.05	0.78	0.75	0.84
20	MCD	4.03 px	12.57 px	12.64 px	12.85 px
	FNR	0.03	0.31	0.31	0.31
	FPR	0.02	0.29	0.31	0.36
30	MCD	4.16 px	15.38 px	15.15 px	15.40 px
	FNR	0.02	0.12	0.13	0.12
	FPR	0.01	0.12	0.11	0.17

**Table 4:** Comparison between YOLOv5 and YOLOv8 for 640×640 resolution.

Threshold	Metrics	AirSim		HML	
		YOLOv5	YOLOv8	YOLOv5	YOLOv8
10	MCD	3.71 px	2.15 px	6.78 px	6.92 px
	FNR	0.07	0.06	0.78	0.83
	FPR	0.05	0.02	0.78	0.84
20	MCD	4.03 px	2.28 px	12.57 px	13.48 px
	FNR	0.03	0.04	0.31	0.35
	FPR	0.02	0.01	0.29	0.36
30	MCD	4.16 px	2.35 px	15.38 px	16.51 px
	FNR	0.02	0.04	0.12	0.09
	FPR	0.01	0.01	0.12	0.10
–	mAP@0.1:0.9	0.89	0.90	0.41	0.49
	mAP@0.5:0.95	0.83	0.88	0.09	0.15

## Data Availability

The data presented in this study are available at http://bytom.pja.edu.pl/drones/ (accessed on 10 July 2023).

## Abbreviations

The following abbreviations are used in this manuscript:

FNR	False Negative Rate
FPR	False Positive Rate
HML	Human Motion Lab
IoU	Intersection over Union
mAP	mean Average Precision
MCD	Mean Centers’ Distance
YOLO	You Only Look Once
